# Yin-Chen-Hao Tang Attenuates Severe Acute Pancreatitis in Rat: An Experimental Verification of *In silico* Network Target Prediction

**DOI:** 10.3389/fphar.2016.00378

**Published:** 2016-10-13

**Authors:** Hong Xiang, Guijun Wang, Jialin Qu, Shilin Xia, Xufeng Tao, Bing Qi, Qingkai Zhang, Dong Shang

**Affiliations:** ^1^College (Institute) of Integrative Medicine, Dalian Medical University Dalian, China; ^2^Department of General Surgery, The First Affiliated Hospital of Jinzhou Medical University Jinzhou, China; ^3^Clinical Laboratory of Integrative Medicine, The First Affiliated Hospital of Dalian Medical University Dalian, China; ^4^College of Pharmacy, Dalian Medical University Dalian, China; ^5^Department of General Surgery, The First Affiliated Hospital of Dalian Medical University Dalian, China

**Keywords:** Yin-Chen-Hao Tang, severe acute pancreatitis, inflammation, apoptosis, network target prediction, PPARγ, NF-κB

## Abstract

Yin-Chen-Hao Tang (YCHT) is a classical Chinese medicine compound that has a long history of clinical use in China for the treatment of inflammatory diseases. However, the efficacy and mechanisms of YCHT for the treatment of severe acute pancreatitis (SAP) are not known. The current study investigated the pharmacological properties of YCHT against SAP and its underlying mechanisms. A computational prediction of potential targets of YCHT was initially established based on a network pharmacology simulation. The model suggested that YCHT attenuated SAP progress by apoptosis inducement, anti-inflammation, anti-oxidation and blood lipid regulation. These effects were validated in SAP rats. YCHT administration produced the following results: (1) significantly inhibited the secretion of pancreatic enzymes and protected pancreatic tissue; (2) obviously increased the number of *in situ* terminal deoxynucleotidyl transferase dUTP nick-end labeling (TUNEL)-positive cells and induced apoptosis; (3) markedly inhibited neutrophil infiltration to the impaired pancreas and reduced the inflammatory reaction; (4) notably enhanced the activities of antioxidant enzymes and decreased the nitric oxide synthase levels; (5) significantly reduced the levels of triglycerides, total cholesterol and low-density lipoprotein and increased high-density lipoprotein; and (6) significantly up-regulated peroxisome proliferator-activated receptor-γ (PPARγ) and down-regulated nuclear factor-kappa B (NF-κB). In summary, these results demonstrated that YCHT attenuated SAP progress by inducing apoptosis, repressing inflammation, alleviating oxidative stress and regulating lipid metabolism partially via regulation of the NF-κB/PPARγ signal pathway.

## Introduction

Acute pancreatitis (AP) is one of the most intractable gastrointestinal disorders that results in hospital admission. AP currently affects 4.9 to 73.4 per 100,000 individual’s annually worldwide (Tenner et al., 2013). According to the revised Atlanta classification from 2012, AP was divided into mild, moderate, or severe. Severe acute pancreatitis (SAP) characterized by rapid change and complicated illness, is constantly accompanied by multi-organ and multi-system impaired or failure (Zhang et al., 2007). Approximately 20% of AP patients deteriorate into SAP with a mortality rate of 10–30% (Famularo et al., 2006; Bakker et al., 2014). The initial phase of SAP is an inappropriate activation of trypsin and a lack of efficient exclusion of active trypsin, which causes pancreatic autodigestion and damage to pancreatic acinar cells that synthesize, store and secrete digestive enzymes (Whitcomb, 2004; Yadav and Lowenfels, 2013). The damaged acinar cells undergo apoptosis or necrosis. Apoptosis maintains the membrane integrity without causing inflammation, and necrosis releases the intracellular contents and triggers an inflammatory cascade (Bhatia, 2004; Gukovskaya and Pandol, 2004). Therefore, the switch from apoptosis to necrosis in acinar cells depends on the severity of pancreatitis.

An acute inflammatory cascade in response to local tissue injury is the second phase of SAP (Peery et al., 2012). The inflammatory cytokines, such as tumor necrosis factor-α (TNF-α), interleukin-1β (IL-1β), and IL-6, which are secreted by activated inflammatory cells, initiate a series of signaling pathways, which results in further damage to tissues (Sanchez-Hidalgo et al., 2005; Lankisch et al., 2015). When inflammation inside the pancreas persists for longer periods, it may spread beyond the pancreas and develop into systemic inflammatory response syndrome (SIRS), multiple organ dysfunction syndrome (MODS), or death in humans and experimental animal, as seen in models (Frossard et al., 2008).

The homeostasis of pro-oxidant and antioxidant molecules in the pancreas is destroyed during the course of SAP by the intrinsic antioxidant systems that cannot immediately eliminate the excessive oxygen free radicals (OFR), which causes oxidative damage in the pancreas (Urunuela et al., 2002; Tsang et al., 2016). Recent studies highlighted the beneficial effects of antioxidant pretreatments, such as superoxide dismutase (SOD) and catalase (CAT), which suggests a role for oxidative stress in the acute inflammatory response, particularly in pancreatic injuries associated with SAP (Esrefoglu, 2012). Oxidative stress also causes hyperlipidemia, which is an early pathological outcome of SAP (Sawalhi et al., 2014). Increased triglycerides (TGs) in the pancreas and peripheral tissues are hydrolyzed by pancreatic lipase, which produces and releases large quantities of free fatty acids (FFAs), which have a direct cytotoxic effect on acinar cells (Nawaz et al., 2015; Gonzalez-Moreno et al., 2016).

Therefore, the intimate links between apoptosis, inflammation, oxidative stress, and lipid deposition play essential roles in SAP. Investigations of the underlying mechanisms of SAP are urgently needed in order to develop novel and effective treatment strategies for this disease.

Meta-analyses and systemic reviews suggest that traditional Chinese medicine (TCM) is a valuable therapeutic strategy and drug resource for patients with SAP (Zhang et al., 2007; Lu et al., 2014). Yin-Chen-Hao Tang (YCHT) is a famous Chinese herbal formula that consists of the following three medicinal herbs: *Artemisia capillaris* Thunb., *Gardenia jasminoides* Ellis, and *Rheum officinale* Baill. YCHT has been used in China as an anti-inflammatory and choleretic agent (Lee et al., 2007). However, no studies have reported the actions of YCHT against SAP to the best of our knowledge. Knowledge of the interaction between complex chemical and biological systems of a syndrome is a major and difficult issue in TCM research (Hopkins, 2007). The integration of network biology and polypharmacology provides ample opportunities for drug targets. This integration may also contribute to the exploration of drug targets and to the identification of potential active ingredients in TCM research (Gao et al., 2016; Hsin et al., 2016).

The present study determined the active ingredients of YCHT using a network pharmacology approach. We also used network target prediction and experimental verification to evaluate the links between herbal ingredients and pharmacological actions.

## Materials and Methods

### Reagents and Materials

The Department of Pharmacy of the First Affiliated Hospital of Dalian Medical University kindly provided YCHT. Sodium taurocholate was obtained from Sigma Chemical Co. (St. Louis, MO, USA). Rat-amylase, lipase, TG, total cholesterol (TC), low-density lipoprotein (LDL) and high-density lipoprotein (HDL) assay enzyme-linked immunosorbent assay (ELISA) kits were purchased from Langdun Biotech (Shanghai, China). Rat C-Reactive Protein (CRP) ELISA kit was purchased from Sigma (Santa Clara, CA, USA). The PowerVision two-step histostaining reagent, CAT, nitric oxide synthase (NOS), and SOD detection kits were obtained from the Nanjing Jiancheng Institute of Biotechnology (Nanjing, China). RNAiso Plus reagent, PrimeScript^®^RT reagent and SYBR^®^ PremixExTaq^TM^II (TliRNaseH Plus) were purchased from TaKaRa Biotechnology Co., Ltd. (Dalian, China). The tissue protein extraction kit was purchased from KEYGEN Biotech. Co., Ltd. (Nanjing, China). TUNEL apoptosis detection and bicinchoninic acid (BCA) protein assay kits were purchased from Beyotime Institute of Biotechnology (Shanghai, China). Anti-caspase-12 and anti-caspase-9 antibodies were purchased from Abcam (Cambridge, UK). The anti-caspase-3 antibody was obtained from Cell Signaling Technology, Inc. (Beverly, MA, USA). Antibodies against rabbit- myeloperoxidase (MPO), peroxisome proliferator-activated receptor-γ (PPARγ) and nuclear factor-kappa B (NF-κB), glyceraldehyde-3-phosphate dehydrogenase (GAPDH)-conjugated Affinipure IgG, horseradish peroxidase-conjugated goat anti-rabbit IgG, and TRITC-conjugated goat anti-rabbit IgG were purchased from Proteintech Group (Chicago, USA).

### Preparation of YCHT

Yin-Chen-Hao Tang was prepared as described in a previous study (Zhang et al., 2016). Briefly, the following three medicinal herbs, *Artemisia capillaris* Thunb., *Gardenia jasminoides* Ellis., and *Rheum officinale* Baill., were mixed at a ratio of 2: 1: 1 by weight. *Artemisia capillaris* Thunb. was mixed with distilled water at 10 times its weight for 1 h, followed by decocting for 40 min. *Gardenia jasminoides* Ellis and *Rheum officinale* Baill. were added separately and boiled for 15 additional min. The filtrate was collected, and the residue was soaked in seven times its weight in water and additionally decocted for 20 min. The two filtrates were combined and concentrated to 0.25 g/ml. The extract was filtered and maintained at 4°C. Lee et al. identified the content of three index components of YCHT using high performance liquid chromatography (HPLC): 0.95% (w/w) geniposide, 0.14% capillarisin and 0.11% emodin (Lee et al., 2009).

### Network Pharmacology Analysis of the Pharmacological Mechanism of YCHT

The active chemical components of YCHT were collected and extracted from PubMed, Web of Science, CNKI^1^, and Wanfang^2^ Databases. After reviewing the articles, we collected 76 compounds that encompassed the main components of YCHT, taking into account the relevant activity reports. We entered the 76 chemical compounds into the TCMSP database to simulate the drug-likeness results with a bioavailability ≥ (OB) 10% and drug-like (DL) ≥ 0.04 and provide further extraction and optimization of the medicinal ingredients (Cheng et al., 2015). Twenty-five of the pharmacological ingredients (**Table 1**; see **Supplementary Figure S1** for the chemical structural formula) that satisfied the above conditions were selected. Then we used DrugBank, TCMID, GeneCards and STITCH databases to validate 89 SAP targets to construct a compound-target network using Cytoscape 3.3.0 software. To explain targets participation in the progression of SAP, we established a network between target and function using Cytoscape 3.3.0. Multiple targets presaged an integral function of YCHT in SAP and shared the synergistic targets of the different compounds.

**Table 1:** Twenty-five active ingredients of YCHT.

Rank	Molecule ID	Ingredient name	OB	DL
M1	MOL001829	Emodin-8-glucoside	10.35	0.8
M2	MOL001414	Saﬄor yellow A	22.75	0.75
M3	MOL008135	3,4-Di-o-caffeoylquinic acid	49.62	0.69
M4	MOL007326	1,3-Dicaffeoylquinic acid	31.76	0.68
M5	MOL004557	Geniposide	14.64	0.44
M6	MOL001668	Geniposidic acid	19.59	0.41
M7	MOL000216	Scopolin	22.91	0.39
M8	MOL003066	Neochlorogenic acid	10.65	0.33
M9	MOL003871	Chlorogenic acid	13.61	0.31
M10	MOL008043	Capillarisin	57.56	0.31
M11	MOL007274	Cirsimaritin	30.35	0.3
M12	MOL002268	Rhein	47.07	0.28
M13	MOL000251	Rhamnocitrin	12.9	0.27
M14	MOL000476	Physcione	22.29	0.27
M15	MOL008046	6-Demethoxycapillarisin	52.33	0.25
M16	MOL000471	Aloe emodin	83.38	0.24
M17	MOL000472	Emodin	24.4	0.24
M18	MOL000492	Catechin	54.83	0.24
M19	MOL001729	Chrysophanol	18.64	0.21
M20	MOL006160	Alizarin	32.67	0.19
M21	MOL001648	Genipin	26.06	0.1
M22	MOL001999	6,7-Dimethylesculetin	74.75	0.09
M23	MOL000414	3,4-Dihydroxycinnamic acid	54.79	0.05
M24	MOL000513	Gallic acid	31.69	0.04
M25	MOL000105	3,4-Dihydroxybenzoic acid	25.37	0.04

### Animals

Sprague-Dawley (SD) rats weighing 250–300 g were obtained from the Dalian Medical University Laboratory Animal Center and housed in a room under a 12-h light-dark cycle with free access to standard laboratory food and water. The ethical committee for Laboratory Animal Care and Use of Dalian Medical University approved the experimental protocol, and all experimental procedures were conducted in accordance with the institutional guidelines for the care and use of laboratory animals.

### SAP Model Establishment

Experimental SAP models were established according to previous reports (Pereda et al., 2004). Briefly, the rats were fasted 12 h with free access to water before the initiation of the experiment. All the animals were anesthetized with intraperitoneal administration of 10% chloral hydrate (0.3 ml/100 g body weight), and the pancreas was fully exposed through a midline incision. The biliopancreatic duct was cannulated through the duodenum, and the hepatic duct was clamped by a small artery clip. Next, the rats were administered a standard retrograde infusion of a freshly prepared 5.0% sodium taurocholate (0.1 ml/100 g body weight) into the biliopancreatic duct. Presenting as controls, sham operation (SO) group received a retrograde infusion of an equivalent volume of sterile saline.

### Experimental groups (Supplementary Figure S2)

SD rats were randomly divided into five groups:

(1) SO group: The SO rats (*n* = 10) underwent the above surgical procedures of SO group.
(2) SAP group: The SAP rats (*n* = 10) underwent the above surgical procedures of SAP group.
(3) SAP + YCHT (4.0 g/kg) group: YCHT (4.0 g/kg) was intragastrically administered to rats in this treatment group (*n* = 10) immediately and 6 h after establishing SAP model.
(4) SAP + YCHT (2.0 g/kg) group: YCHT (2.0 g/kg) was intragastrically administered to rats in this treatment group (*n* = 10) immediately and 6 h after establishing SAP model.
(5) SAP + YCHT (1.0 g/kg) group: YCHT (1.0 g/kg) was intragastrically administered to rats in this treatment group (*n* = 10) immediately and 6 h after establishing SAP model.

Pancreatic head samples were obtained 12 h after duct infusion (Abliz et al., 2015). Blood samples from the abdominal aorta were collected for biochemical analyses. Specimens of the pancreatic head were fixed in 4% paraformaldehyde (PFA, Sigma–Aldrich) and embedded in paraffin for histopathological, immunohistochemical and apoptosis examinations. A small portion (<1 mm^3^) of tissue was excised from the pancreatic head and fixed in 2% glutaraldehyde for transmission electron microscopy (TEM). The other portion of pancreatic samples were immediately frozen and maintained at -80°C for real-time PCR and western blotting analyses.

### Biochemical Analysis

Plasma levels of amylase, lipase, TG, TC, LDL, and HDL were measured using ELISA kits (Langdun Biotech, China) according to the manufacturers’ instructions. C-Reactive Protein (CRP) level in rats plasma was detected by using rat-CRP ELISA kit (Sigma, USA). In addition, SOD, CAT and NOS were measured using spectrophotometry following the manufacturers’ instructions. Blood glucose, calcium, and lactate dehydrogenase (LDH) were measured using Automatic Biochemical Analyzer (Hitachi 7600-020, Japan). Leukocytes and hematocrit were measured using Automatic Blood Cell Analyzer (sysmex XE-5000, Japan).

### Histopathological Examination

Paraformaldehyde-treated tissue samples from each group were embedded in paraffin wax. Pancreatic sections (5 μm) were dewaxed in graded alcohols, stained with hematoxylin and eosin (H&E) and examined under a light microscope (Leica DM4000B, Germany) at 200 × magnification. Images were obtained, and the effects of YCHT on pancreatic tissue damage were evaluated by a pancreatic specialist and an experienced pathologist blinded to the experimental design. We used the scoring system in Supplemental Table S1, and the grading was calculated as previously reported (Yao et al., 2015). The extent of acinar cell ghosts, vacuolization, interstitial edema and interstitial inflammation affected the pancreas (0 normal and 4 severe), giving a maximum score of 12.

### Ultrastructural Examination

Fixed specimens of pancreas were washed three times (each for 15 min) in 0.1 M sodium cacodylate buffer and fixed in 1% osmium tetroxide for 2 h. The samples were dehydrated in gradient ethanol solutions and embedded in Epon 812 resin. The pretreated samples were sliced on an ultramicrotome and collected on copper grids and then counterstained with uranyl acetate and lead citrate. The obtained sections were counterstained and examined using a TEM (JEM-2000EX, JEDL, Japan) at 25,000 × magnification.

### *In situ* Terminal Deoxynucleotidyl Transferase dUTP Nick-End Labeling (TUNEL) Assay

Paraffin sections were dewaxed, and the detection of apoptosis in pancreatic tissue was performed using a TUNEL assay according to the manufacturer’s instructions. Briefly, a fluorescein (red)-labeled dUTP solution was added to the sections, incubated at 37°C for 1 h, and washed with phosphate buffer saline (PBS). The images were photographed using a fluorescence microscopy (Olympus BX63, Japan) at 200 × magnification. The apoptosis rate was estimated as the average number of positive cells (red spots) in the fluorescent images.

### Detection of MPO in Pancreatic Tissues

Immunofluorescence staining was performed in pancreas sections using the standard procedures described above (Xiang et al., 2016). Slides were incubated with diluted MPO antibodies (1:100) and incubated with TRITC-conjugated goat anti-rabbit IgG (H + L) for 1 h at 37°C. Digital images were obtained using an Olympus BX63 fluorescent microscope (Olympus BX63, Japan) at 200 × magnification. The immunofluorescence staining results were quantified using fluorescence intensity.

The PowerVision two-step histostaining reagent was used to calculate the relative positive expression of MPO in pancreatic tissues. Briefly, pancreas sections were dewaxed in graded alcohol and rinsed in tap water. Endogenous peroxidase activity was inhibited via incubation with 3% H_2_O_2_. Sections were blocked with 5% albumin from bovine serum and incubated for 10 min. An MPO polyclonal antibody (1: 100) was added and incubated overnight at 4°C. Slides were washed in PBS (three times for 15 min) and incubated with a secondary antibody for 60 min at 37°C. Images were acquired using a light microscope (Leica DM4000B, Germany) at 200 × magnification. The immunohistochemical staining results were quantified using integral optic density (IOD). Fluorescent intensity and IOD of the images were counted using the Image-Pro Plus 6.0 software package (Media Cybernetics, USA).

### Quantitative Real-Time PCR Analysis for Inflammatory Cytokines

Total RNA from pancreatic tissues was extracted using RNAiso Plus reagent according to the manufacturer’s protocol. RNA was reverse-transcribed using a PrimeScript^®^ RT reagent kit in a TC-512 PCR system (TECHNE, Stone, UK), and the mRNA expression levels was quantified using real-time PCR with SYBR^®^ Premix ExTaq^TM^II (TliRNaseH Plus) in an ABI 7500 Real-Time PCR System (Applied Biosystems, Foster City, CA, USA). Supplemental Table S2 lists the primers that were used. Amplification was performed at 95°C for 30 s, followed by 40 cycles of denaturing at 95°C for 5 s and annealing at 60°C for 34 s. A no-template control was analyzed in parallel for each gene, and the GAPDH gene was used as a housekeeping gene. The unknown template was calculated using a standard curve for quantitative analysis.

### Western Blotting Assay

Protein samples were extracted from the pancreatic head in different groups using appropriate cold lysis buffer containing 1 mM phenylmethylsulfonyl fluoride (PMSF) based on the instructions of manufacturer. Then, the samples were separated by SDS-PAGE (10–15%) and transferred onto a PVDF membrane (Millipore, USA). Membranes were blocked and individually incubated at 4°C overnight with the following antibodies: caspase-12, caspase-9, caspase-3, PPARγ and NF-κB (1:1000 dilution). Membranes were incubated at room temperature with an appropriate secondary antibody, and an enhanced chemiluminescence (ECL) method was used to visualize proteins. Protein bands were imaged in a ChemiDoc XRS bioimaging system (Bio-Rad, USA). Bands were normalized to GAPDH to ensure equal protein loading.

### Statistical Analysis

The measurement data are expressed as the mean ± standard deviation (SD) and analyzed using one-way repeated-measures ANOVA followed by the least significance difference (LSD) test in order to detect differences between groups. Differences with a value *P* < 0.05 or *P* < 0.01 were considered as statistically significant. All statistical analyses were performed using SPSS 18.0 software (SPSS Inc., Chicago, IL, USA).

## Results

### Prediction Analysis of YCHT Attenuates SAP Based on Network Pharmacology

**FIGURE 1:**
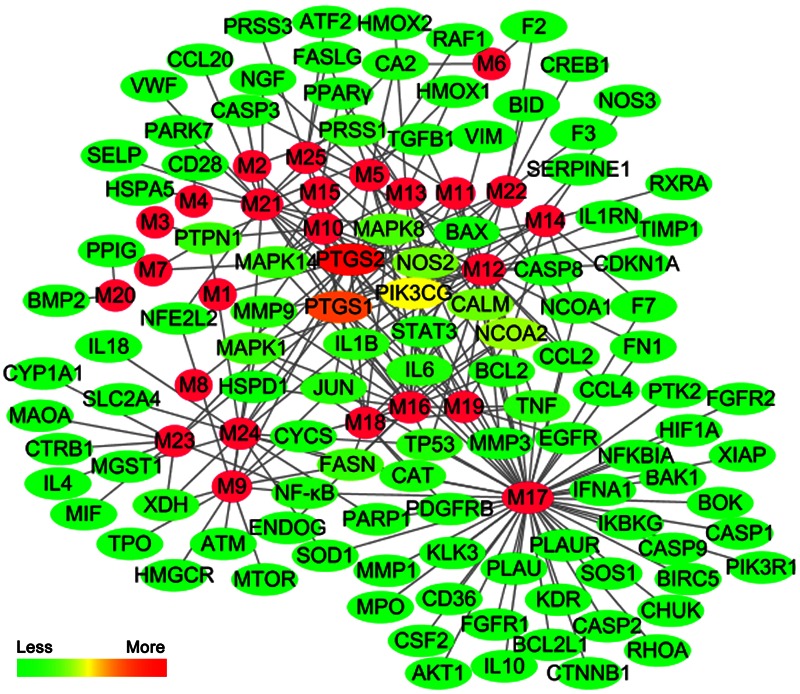
**The compound-target network**.

**FIGURE 2:**
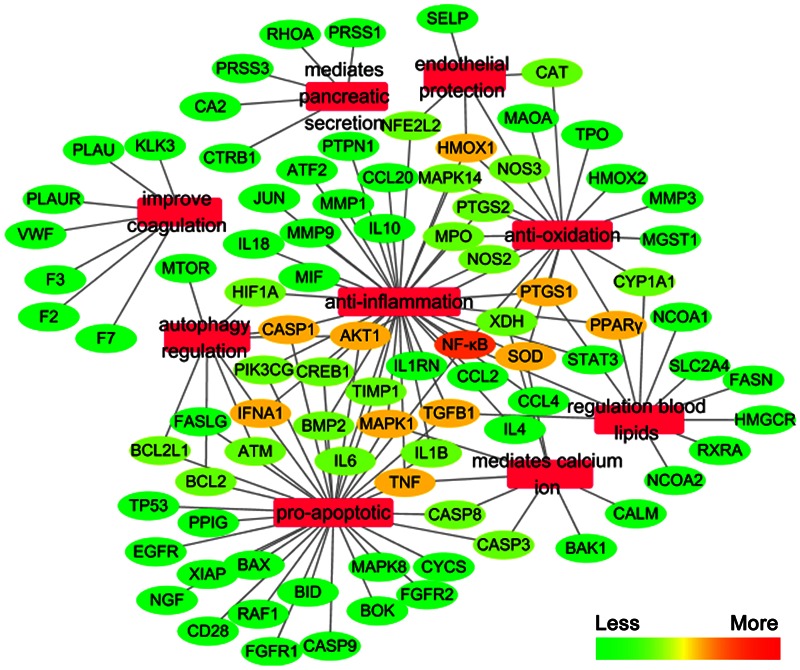
**The target-function network**.

We constructed the component-target network (C-T network, **Figure 1**) based on data mining, which consists of 25 compounds and 89 candidate targets. This network had 114 nodes and 237 edges, in which pink circles and different colors-ellipses correspond to active components and targets, respectively. In the relationships between compounds and targets, for those active compounds, M17 exhibits the highest number of target candidate target interaction (degree = 55), followed by M12 (degree = 24), M16 and M19 (degree = 19) and so on, these emphasize the multi-target properties of ingredients, which is the basis of the action mode of TCM compounds. To explain targets participation in the progression of SAP, target and function network was constructed by linking the potential targets and their corresponding nine functional annotations (T-F network, **Figure 2**). As for the candidate targets, NF-κB shows the highest degree (degree = 4), with PPARγ (Peroxisome proliferator activated receptor gamma, degree = 3) behind it, which demonstrate the potential therapeutic effect of YCHT on SAP through modulating these relevant proteins.

### YCHT Attenuated SAP Injury in Rats

The clinical chemistry workup indexes including blood glucose, calcium, LDH, CRP, leukocytes, and hematocrit are the important criterions for judging SAP. Therefore, we detected these parameters and found that SAP induced the decrease of calcium and the increase of blood glucose, LDH, CRP, leukocytes and hematocrit in rats. However, compared to the SAP group, YCHT could notably improve these indexes with a dose-dependent manner (**Table 2**).

**Table 2:** The effects of YCHT on the biochemical parameters in SAP rats.

Parameters	SO	SAP	YCHT (4.0 g/kg)	YCHT (2.0 g/kg)	YCHT (1.0 g/kg)
Blood glucose (mmol/L)	4.82 ± 1.31	11.81 ± 3.24^∗∗^	7.21 ± 0.97^##^	8.59 ± 1.13^##^	9.18 ± 0.98^##^
Calcium (mmol/L)	2.52 ± 0.38	1.64 ± 0.38^∗∗^	2.10 ± 0.25^##^	1.90 ± 0.25	1.72 ± 0.32
LDH (U/L)	317.57 ± 93.37	2019.00 ± 634.00^∗∗^	669.50 ± 194.13^##^	855.43 ± 203.16^##^	1213.88 ± 186.61^##^
CRP (ng/ml)	680.22 ± 67.65	1018.70 ± 111.04^∗∗^	764.74 ± 155.32^##^	828.06 ± 196.10^#^	890.84 ± 146.21
Leukocytes (× 10^9^/L)	7.11 ± 1.42	11.17 ± 2.13^∗∗^	7.81 ± 1.05^##^	9.83 ± 0.77	10.11 ± 1.89
Hematocrit	0.49 ± 0.02	0.60 ± 0.08^∗∗^	0.53 ± 0.05^#^	0.54 ± 0.04	0.59 ± 0.04

*The data are presented as the mean ± SD, ^∗∗^*P* < 0.01 versus SO; ^#^*P* < 0.05 versus SAP, ^##^*P* < 0.01 versus SAP.*

**FIGURE 3:**
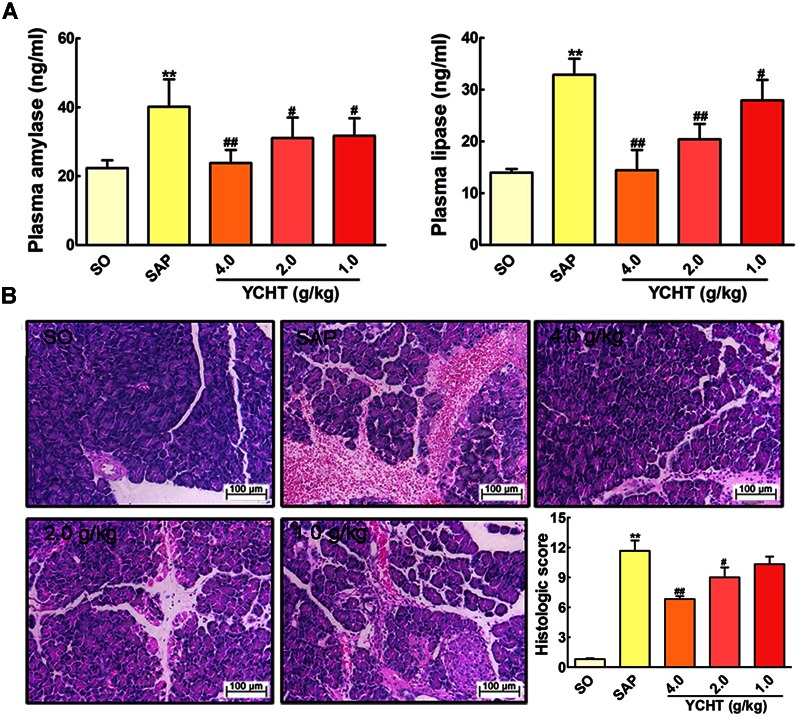
**Yin-Chen-Hao Tang (YCHT) attenuated pancreas damage in SAP rats. (A)** Effects of YCHT on the plasma levels of amylase and lipase in SAP rats. **(B)** Effects of YCHT on H&E staining of pancreatic tissue in SAP rats. Images are presented at 200 × magnification. The data are presented as the mean ± SD, *n* = 10. ^∗∗^*P* < 0.01 versus SO; ^#^*P* < 0.05 versus SAP, ^##^*P* < 0.01 versus SAP.

The direct trigger of SAP relates to trypsin-mediated pancreatic autodigestion (Whitcomb, 2004). Amylase and lipase are important pancreatic enzymes and the biochemical hallmarks of SAP (Famularo et al., 2006; Kosekli et al., 2016). ELISA indicated that plasma amylase and lipase levels in the SAP group were approximately increased by 2 times compared with the SO group (*P* < 0.01) (**Figure 3A**). However, YCHT at 4.0, 2.0, and 1.0 g/kg doses decreased plasma amylase and lipase levels in a dose-dependent manner compared to the SAP group.

To examine the effect of YCHT on the severity of SAP, we investigated the histological architecture of the pancreas against a sodium taurocholate infusion challenge. In SO rat, no histological abnormalities were observed in the pancreas. However, in SAP group, H&E revealed tissue damage characterized by acinar cell vacuolization, interstitial edema, hemorrhage and necrosis, and leukocyte infiltration; the histological score was obviously increased as compared with the SO group (**Figure 3B**). YCHT dramatically attenuated these morphological changes at 4.0, 2.0, and 1.0 g/kg, thus decreasing pancreatic pathological scores.

### YCHT Improved Acinar Cellular Ultrastructure Injury in Rats

**FIGURE 4:**
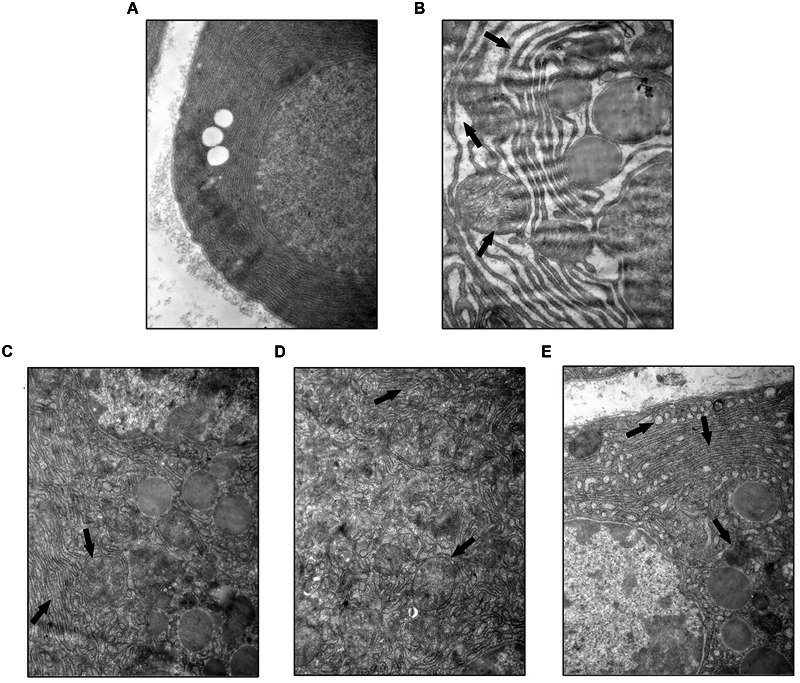
**Yin-Chen-Hao Tang attenuated cellular structural changes in pancreas of SAP rats.** Typical images of cell ultrastructure in the SO **(A)**, SAP **(B)**, 4.0 g/kg YCHT **(C)**, 2.0 g/kg YCHT **(D)** and 1.0 g/kg YCHT **(E)** groups. Images are presented at 25,000×magnification.

Ultrastructural heterogeneity of acinar cells in SAP rat observed by TEM was mainly caused by pronounced changes in the architectonics of the granular cytoplasmic reticulum. Acinar cells in the SO group exhibited a regular cellular structure with an abundant cytoplasmic reticulum in the basal zones (**Figure 4A**). However, the granular cytoplasmic reticulum in acinar cells was significantly unevenly dilated in SAP rats, with an accumulation of dense materials and autophagic vacuoles. Adverse changes in the solitary mitochondria and cytoplasmic vacuoles were seen in the basal zones of acinar cells (**Figure 4B**, the black arrow). YCHT administration (4.0, 2.0, and 1.0 g/kg) notably improved these ultrastructural alterations in a dose-dependent manner (**Figures 4C–E**, the black arrow).

### YCHT Induced Cell Apoptosis in SAP Rats

**FIGURE 5:**
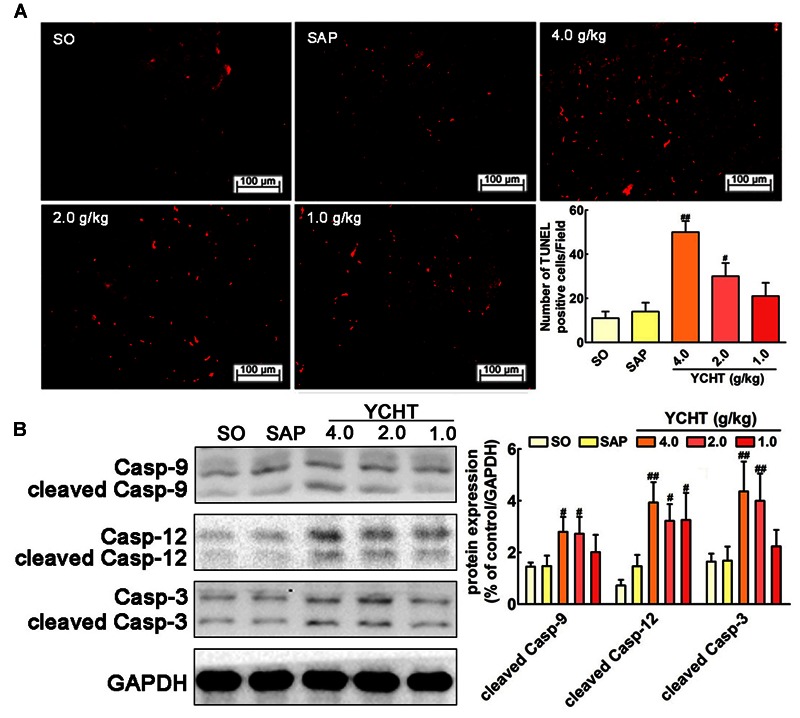
**Yin-Chen-Hao Tang induced cells apoptosis in the pancreas of SAP rats. (A)** Effects of YCHT on cell apoptosis (red) staining of pancreatic tissue in SAP rats by TUNEL detection (*n* = 6). **(B)** Effects of YCHT on the protein expression of cleaved caspase-9, caspase-12 and caspase-3 (*n* = 3). Images are presented at 200× magnification. The data are presented as the mean ± SD, ^#^*P* < 0.05 versus SAP, ^##^*P* < 0.01 versus SAP. The original bands of western blot were shown in **Supplementary Figure S3**.

We evaluated the effect of YCHT in order to assess whether it could promote acinar cell apoptosis. As shown in **Figure 5A**, the number of TUNEL-positive cells exhibiting red fluorescence was markedly higher in YCHT groups than the SAP group, indicating that apoptosis were promoted by YCHT. YCHT also up-regulated the protein expression of cleaved caspase-9, cleaved caspase-12 and cleaved caspase-3 compared to the SAP group (**Figure 5B**).

### YCHT Improved SAP Induced-Inflammation in Rats

**FIGURE 6:**
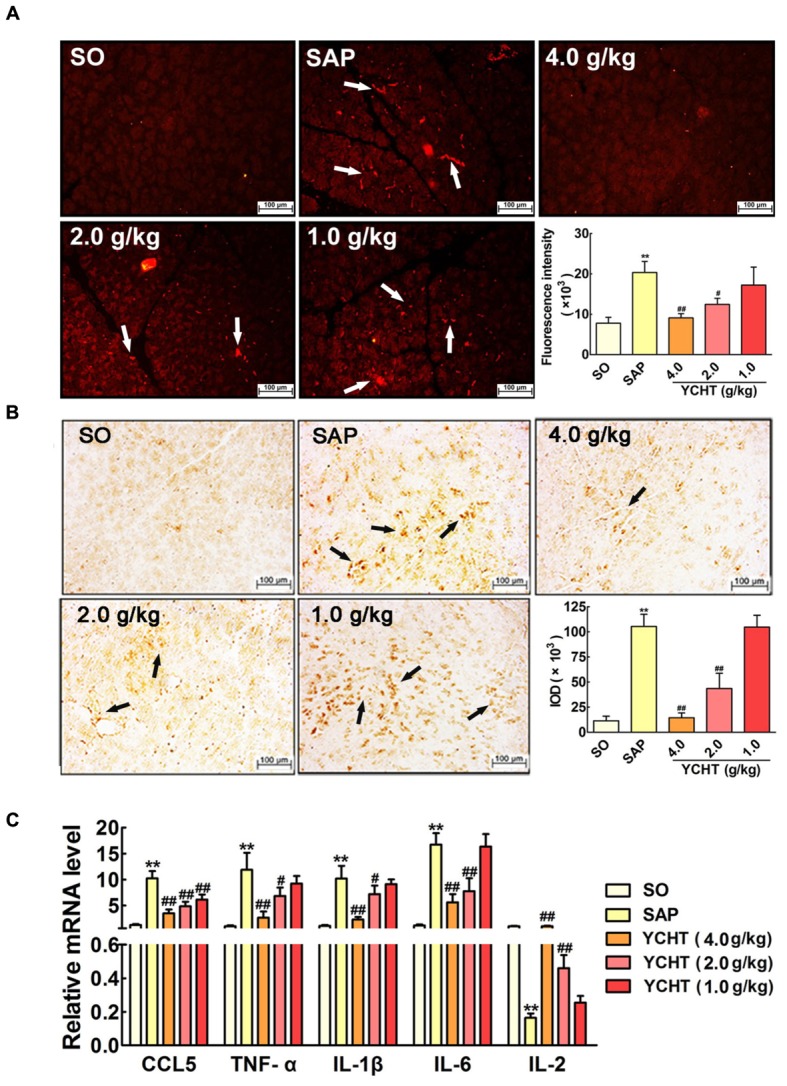
**Yin-Chen-Hao Tang reduced neutrophil infiltration and inflammatory mediator release in SAP rats. (A)** Effects of YCHT on MPO-immunopositive (red) staining area of pancreatic tissue in SAP rats using immunofluorescence detection (*n* = 6). **(B)** Effects of YCHT on MPO-immunopositive (brown) staining area of pancreatic tissue in SAP rats using immunohistochemical detection (*n* = 6). **(C)** Effects of YCHT on the inflammatory mediators CCL5, TNF-α, IL-1β, IL-6 and IL-2 mRNA levels of SAP rats (*n* = 3). Images are presented at 200 × magnification. The data are presented as the mean ± SD, ^∗∗^*P* < 0.01 versus SO; ^#^*P* < 0.05 versus SAP, ^##^*P* < 0.01 versus SAP.

Myeloperoxidase is an indicator of neutrophil sequestration in the pancreas after SAP insult (Xiang et al., 2016). Immunofluorescence indicated that SAP rats exhibited a larger MPO-positive area (red area, as indicated by the white arrow) in pancreas tissue than the SO group (**Figure 6A**). YCHT obviously down-regulated MPO expression, as exhibited by the decreased fluorescence intensity compared with the SAP group. Immunohistochemical analyses also demonstrated that YCHT notably decreased MPO expression (brown area, as indicated by the black arrow), as exhibited by the decrease in IOD compared with the SAP group (**Figure 6B**).

Severe acute pancreatitis is an acute inflammatory disorder that results in increased production of inflammatory mediators. As shown in **Figure 6C**, the mRNA levels of pro-inflammatory cytokines, including chemokine ligand 5 (CCL5), TNF-α, IL-1β, and IL-6, were notably increased in the SAP group, and the level of the anti-inflammatory mediator IL-2 was obviously decreased in the SAP group compared with the SO group (*P* < 0.01). YCHT treatment significantly restored the levels of these mediators to the SO group at 4.0, 2.0, and 1.0 g/kg.

### YCHT Improved SAP-Induced Oxidative Stress and Lipid Metabolism in Rats

**FIGURE 7:**
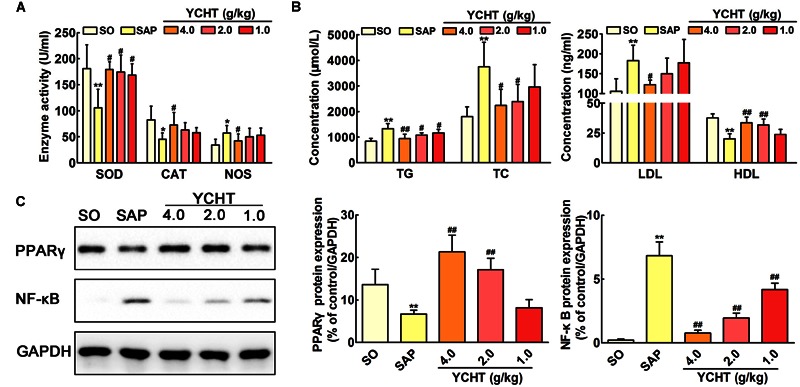
**Yin-Chen-Hao Tang regulated oxidative stress, lipid dysmetabolism and cell apoptosis in SAP rats. (A)** Effects of YCHT on plasma redox parameters in SAP rats (*n* = 10). **(B)** Effects of YCHT on the plasma lipid profile in SAP rats (*n* = 10). **(C)** Effects of YCHT on the protein expression of PPARγ and NF-κB in SAP rats (*n* = 3). The data are presented as the mean ± SD, ^∗^*P* < 0.05 versus SO, ^∗∗^*P* < 0.01 versus SO; ^#^*P* < 0.05 versus SAP, ^##^*P* < 0.01 versus SAP. The original bands of western blot were shown in **Supplementary Figure S4**.

**Figure 7A** shows the parameters of redox behavior in rats. Plasma SOD and CAT levels were markedly decreased, and the plasma NOS level was increased in the SAP group compared with SO rats. YCHT administration yielded significantly higher activity of antioxidant enzymes and modified oxidative stress marker. YCHT up-regulated SOD and CAT levels and down-regulated the NOS level compared with the SAP group (*P* < 0.05).

Increased levels of TG, TC, and LDL and decreased HDL were observed in SAP rats, and YCHT significantly reversed these alterations (**Figure 7B**). Thus, YCHT administration mediated the lipid profile, especially reducing TG, TC, and LDL, and increasing HDL in SAP rats. Our data suggest that YCHT improves the serum lipid profile that associates with the pathogenesis of SAP.

### YCHT Improved PPARγ and NF-κB Protein Expression in SAP Rats

Western blotting analysis was used to evaluate PPAR-γ and NF-κB protein levels in the pancreas. As shown in **Figure 7C**, PPAR-γ expression was notably down-regulated and NF-κB expression was markedly up-regulated in SAP rats following sodium taurocholate infusion. As expected, YCHT administration significantly increased PPAR-γ level and decreased the NF-κB level compared to the SAP group.

## Discussion

The high morbidity and mortality of SAP and the powerlessness of current therapy in dealing with this disease cause it an urgent requirement for us to seek efficient therapeutic methods (Qiong et al., 2005). The efficacy of TCM treating complex diseases has been verified through 1000s of years’ practice (Berger and Iyengar, 2009; Schenone et al., 2013). Our findings in the current experiments indicated that YCHT decreased plasma amylase and lipase levels in a dose-dependent manner. The results of clinical chemistry workup indexes including calcium, blood glucose, LDH, CRP, leukocytes, and hematocrit suggested YCHT notably improved these parameters in a dose-dependent manner. The H&E staining and TEM results demonstrated that YCHT notably attenuated SAP-induced morphological changes and ultrastructural alterations. To the best of our knowledge, the present study, for the first time, demonstrated that YCHT attenuated SAP injury in an established rat SAP model.

Network pharmacology provides new methods in TCM research to explore the multi-target effects of drug action and theoretical thinking of biological network performances (Cheng et al., 2015; Chen et al., 2016). To better reveal the molecular mechanism of YCHT treatment of SAP, the C-T and T-F network of YCHT were established based on the network pharmacology analysis, which consists of 25 compounds, 89 candidate targets and their corresponding nine functional annotations, respectively. The analytical result displayed that multiple targets hit by the same compound or different compounds acting on the same targets get more opportunities to influencing the whole balance of networks, which make the YCHT therapy more effective. We supposed that YCHT prevents SAP may be primarily by improving cell apoptosis, inflammatory reaction, oxidative stress, and lipid metabolism.

Previous researchers indicated that there was a negative correlation between the rate of apoptosis of pancreatic cells and SAP severity. Most acinar cells undergo necrosis during the early stage of SAP, and only a small number of cells are apoptotic (Bhatia, 2004; Gukovskaya and Pandol, 2004). The death of pancreatic cells in SAP involves the formation of apoptosis via the mitochondrial pathway (i.e., caspase-9 cascade) and endoplasmic reticulum pathway (i.e., caspase-12 cascade) (Fu et al., 2016; Liu et al., 2016). YCHT treatment in the current study significantly induced cell apoptosis in pancreatic tissues as measured using TUNEL detection and up-regulated apoptosis-associated caspase expression (cleaved caspases-9, -12, and -3) compared to the SAP group. These results suggest that YCHT alleviates SAP via the induction of cellular apoptosis.

The intracellular contents that are released from necrotic acinar cells promote leukocyte infiltration into the damaged pancreas and induce an acute inflammatory response that results in substantial tissue damage (Frossard et al., 2008). MPO is a classical biomarker of neutrophil activation that plays an important role in inflammation, especially in the occurrence and development of SAP (Yang et al., 2015; Xiang et al., 2016). Immunofluorescence and immunohistochemistry results demonstrated that YCHT treatment inhibited MPO protein expression. The levels of pro-inflammatory cytokines, including CCL5, IL-1β, IL-6, and TNF-α, were obviously increased in SAP rats, and the anti-inflammatory cytokine IL-2 decreased. CCL5 is a member of a chemokine family that plays an important role in leukocyte chemotaxis (e.g., monocytes and neutrophils) from the blood circulation to the site of tissue injury (Moreno et al., 2006). TNF-α as a pleiotropic cytokine that mediates the synthesis and release of other pro-inflammatory mediators and leukocyte adhesion. IL-1β and IL-6 estimate the degree of injury to local tissue and the severity of SAP (Granger and Remick, 2005). This study measured these inflammatory responses and found that YCHT decreased the mRNA levels of CCL5, TNF-α, IL-1β, and IL-6 and increased IL-2. These results confirmed that YCHT attenuated SAP by decreasing neutrophil sequestration and the inflammatory response.

A cellular redox imbalance caused by a combination of increased NOS activity and decreased antioxidant enzymes activity, e.g., SOD and CAT, plays a significant role in the pathogenesis of inflammation, including SAP. Oxidative stress and inflammation influence and aggravate each other in SAP (Urunuela et al., 2002; Esrefoglu, 2012; Tsang et al., 2016). YCHT administration in the present study increased the activities of SOD and CAT and decreased NOS, which resulted in a prominent elevation of total antioxidant capacity.

Convincing evidence emphasizes the significance of hyperlipidemia in the increased risk of SAP as a promoter of further SAP development (Carr et al., 2016; Mikolasevic et al., 2016). Our results indicated that YCHT decreased TG, TC and LDL and increased HDL, which suggests that YCHT improved the serum lipid metabolism alterations that are associated with the pathogenesis of SAP.

NF-κB was shown to be a potential therapeutic target for SAP in the present study. The activation of NF-κB is a central and early inflammatory event in SAP (Jakkampudi et al., 2016). Briefly, NF-κB exerts an anti-apoptotic effect on the process of programmed cell death in acinar cells, which results in decreased apoptosis and increased necrosis. NF-κB links the initial acinar insult to the systemic inflammatory response. The inflammatory factors released from the necrotic acinar cells further cause the production of the initial signal, which leads to the infiltration of neutrophils into the pancreas and enhances the inflammatory cascade (Wang et al., 2013; Xu et al., 2013). Oxidative stress and hyperlipidemia are also important mediators that initiate an intra-acinar NF-κB-mediated inflammatory cascade (Jakkampudi et al., 2016). Nuclear factor PPAR-γ negatively modulates the expression of pro-inflammatory genes and the NF-κB protein and plays an important role in the inflammatory resolution of SAP (Wan et al., 2008). Western blotting revealed that YCHT markedly down-regulated NF-κB protein expression and up-regulated PPAR-γ protein expression in the present study. Therefore, the anti-inflammatory action of YCHT may result partially from inhibition of the NF-κB signal pathway.

## Conclusion

**FIGURE 8:**
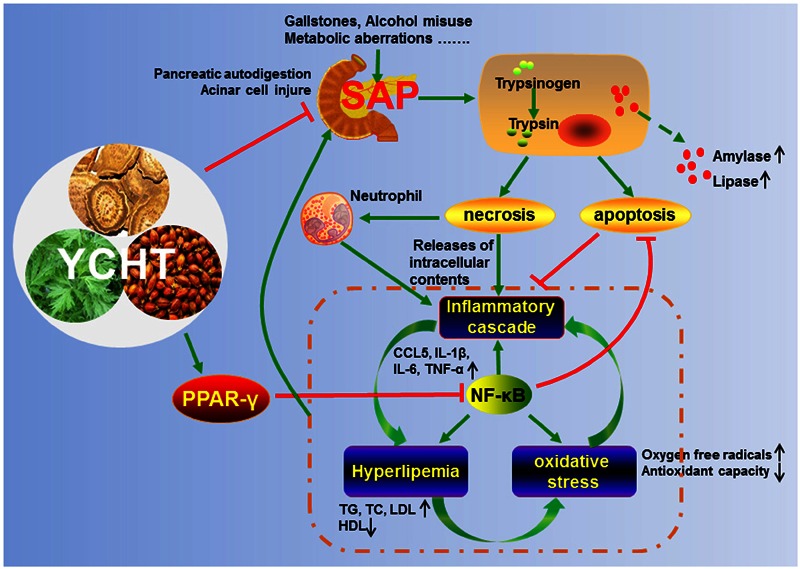
**Proposed model for the protective effects of YCHT against SAP**.

Our findings demonstrated that YCHT attenuated SAP. The underlying molecular mechanisms may involve pro-apoptosis, anti-inflammation, anti-oxidation and regulation of the lipid profile, partially via regulation of the PPARγ/NF-κB signal pathway (**Figure 8**). Our results support the ability of YCHT to inhibit SAP and offer a potential therapy for SAP.

## Author Contributions

HX is the first author and performed all the *in vivo* experiments like animal experiments setup, biochemical estimation, and tissue processing etc. GW, JQ, SX, and XT helped the first author in different steps of experiments. BQ and QZ helped in literature survey and manuscript preparation. Prof. DS contributed toward study design, experimental setup, results supervision, and manuscript correction.

## Conflict of Interest Statement

The authors declare that the research was conducted in the absence of any commercial or financial relationships that could be construed as a potential conflict of interest.

## Footnotes

1 http://www.cnki.net/

2 http://www.wanfangdata.com.cn/

## Supplementary Material

FIGURE S1 **The pharmacological ingredients of YCHT**.

FIGURE S2 **Experimental protocol of YCHT attenuates SAP**.

FIGURE S3 **The original bands of western blot results in Figure 5**.

FIGURE S4 **The original bands of western blot results in Figure 7**.
