# Corrigendum: Yin-Chen-Hao Tang Attenuates Severe Acute Pancreatitis in Rat: An Experimental Verification of *In silico* Network Target Prediction

**DOI:** 10.3389/fphar.2018.01203

**Published:** 2018-10-17

**Authors:** Hong Xiang, Guijun Wang, Jialin Qu, Shilin Xia, Xufeng Tao, Bing Qi, Qingkai Zhang, Dong Shang

**Affiliations:** ^1^College (Institute) of Integrative Medicine, Dalian Medical University, Dalian, China; ^2^Department of General Surgery, The First Affiliated Hospital of Jinzhou Medical University, Jinzhou, China; ^3^Clinical Laboratory of Integrative Medicine, The First Affiliated Hospital of Dalian Medical University, Dalian, China; ^4^College of Pharmacy, Dalian Medical University, Dalian, China; ^5^Department of General Surgery, The First Affiliated Hospital of Dalian Medical University, Dalian, China

**Keywords:** Yin-Chen-Hao Tang, severe acute pancreatitis, inflammation, apoptosis, network target prediction, PPARγ, NF-κB

In the original article, there was a mistake in Figure 6A. The wrong slide of microscopy provided in this figure. The corrected Figure 6 appears below. The authors apologize for this error and state that this does not change the scientific conclusions of the article in any way.

**Figure 6:**
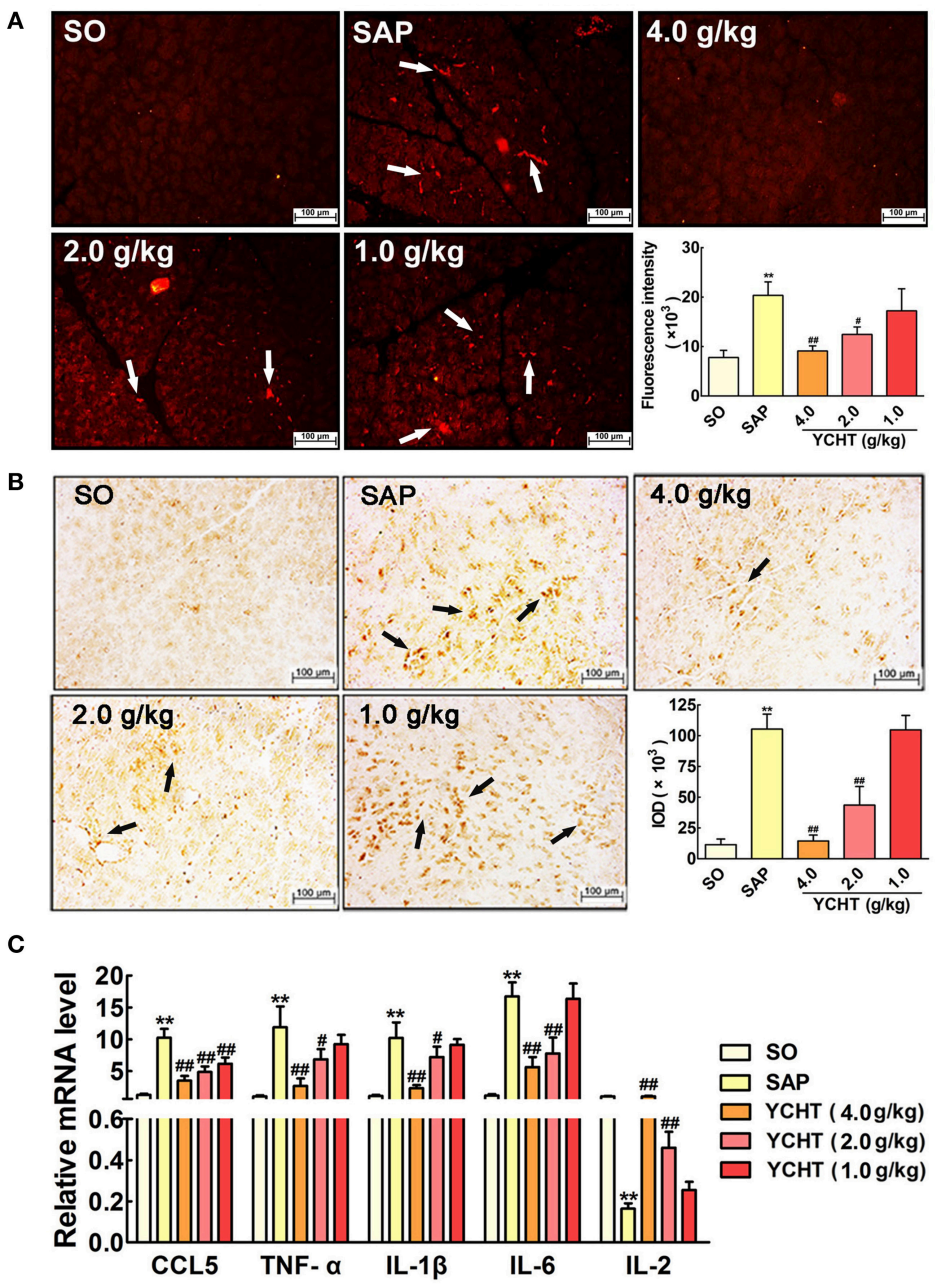
Yin-Chen-Hao Tang reduced neutrophil infiltration and inflammatory mediator release in SAP rats. **(A)** Effects of YCHT on MPO-immunopositive (red) staining area of pancreatic tissue in SAP rats using immunofluorescence detection (*n* = 6). **(B)** Effects of YCHT on MPO-immunopositive (brown) staining area of pancreatic tissue in SAP rats using immunohistochemical detection (*n* = 6). **(C)** Effects of YCHT on the inflammatory mediators CCL5, TNF-α, IL-1β, IL-6 and IL-2 mRNA levels of SAP rats (*n* = 3). Images are presented at 200 × magnification. The data are presented as the mean ± SD, ^**^P < 0.01 versus SO; ^#^*P* < 0.05 versus SAP, ^##^*P* < 0.01 versus SAP.

The original article has been updated.

## Conflict of interest statement

The authors declare that the research was conducted in the absence of any commercial or financial relationships that could be construed as a potential conflict of interest.

