# Supplementary material for: Yin-Chen-Hao Tang Attenuates Severe Acute Pancreatitis in Rat: An Experimental Verification of In silico Network Target Prediction
Source: Front Pharmacol. 2016 Oct 13;7:378. doi: 10.3389/fphar.2016.00378 (PMC5061810; doi:10.3389/fphar.2016.00378)
Supplement: Supplementary file 1 [file Table_1.DOC]

**Supplemental Table 1.** Histological scoring for acute pancreatitis.

| Condition | Score | Description |
| --- | --- | --- |
| Edema | 0  1  2  3  4 | Absent  Diffuse expansion of interlobular septa  1 + diffuse expansion of interlobular septa  2 + diffuse expansion of interlobular septa  3 + diffuse expansion of intercellular septa |
| Inflammation (%) | 0  1  2  3  4 | Absent  Around ductal margin  In parenchyma (< 50 of lobules)  In parenchyma (51-75 of lobules)  In parenchyma (> 75 of lobules) |
| Vacuolization (%) | 0  1  2  3  4 | Absent  Periductal (<5)  Focal (5-20)  Diffuse (21-50)  Severe (> 50) |
