# Supplementary material for: Yin-Chen-Hao Tang Attenuates Severe Acute Pancreatitis in Rat: An Experimental Verification of In silico Network Target Prediction
Source: Front Pharmacol. 2016 Oct 13;7:378. doi: 10.3389/fphar.2016.00378 (PMC5061810; doi:10.3389/fphar.2016.00378)
Supplement: Supplementary file 2 [file Table_2.DOC]

**Supplemental Table 2.** The primer sequences used for real-time PCR assay

| Gene | GenBank accession | Primers (5'-3') |
| --- | --- | --- |
| IL-6 | NM_012589.2 | Forward:CTGATTGTATGAACAGCGATGATG  Reverse:GGTAGAAACGGAACTCCAGAAGAC |
| IL-1β | NM_012589.2 | Forward: TCAGGAAGGCAGTGTCACTCA  Reverse: CATCATCCCACGAGTCACAGA |
| TNF-α | NM_012675.3 | Forward: CAAGAGCCCTTGCCCTAAGG  Reverse:CGGACTCCGTGATGTCTAAGTACTT |
| CCL5 | NM_031116.3 | Forward: GACACCACTCCCTGCTGCTT  Reverse: CTTCTCTGGGTTGGCACACA |
| IL-2 | NM_053836.1 | Forward: CCATGATGCTCACGTTTAAATTTT  Reverse: TGAAATTTCCAGCGTCTTCCA |
