# Supplementary figures and images for: Yin-Chen-Hao Tang Attenuates Severe Acute Pancreatitis in Rat: An Experimental Verification of In silico Network Target Prediction
Source: Front Pharmacol. 2016 Oct 13;7:378. doi: 10.3389/fphar.2016.00378 (PMC5061810; doi:10.3389/fphar.2016.00378)

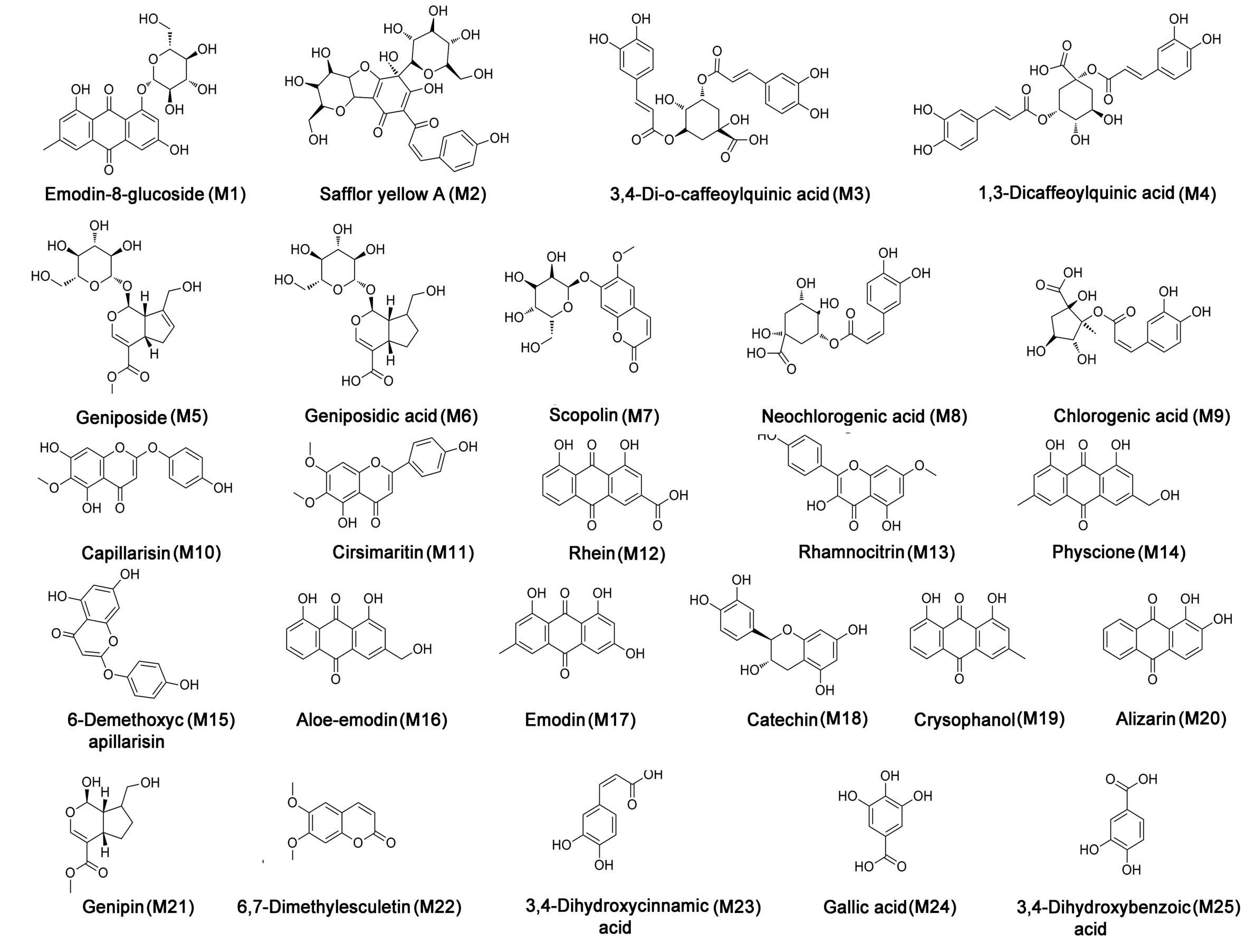

Supplement: FIGURE S1 — The pharmacological ingredients of YCHT. [file Image_1.JPEG]

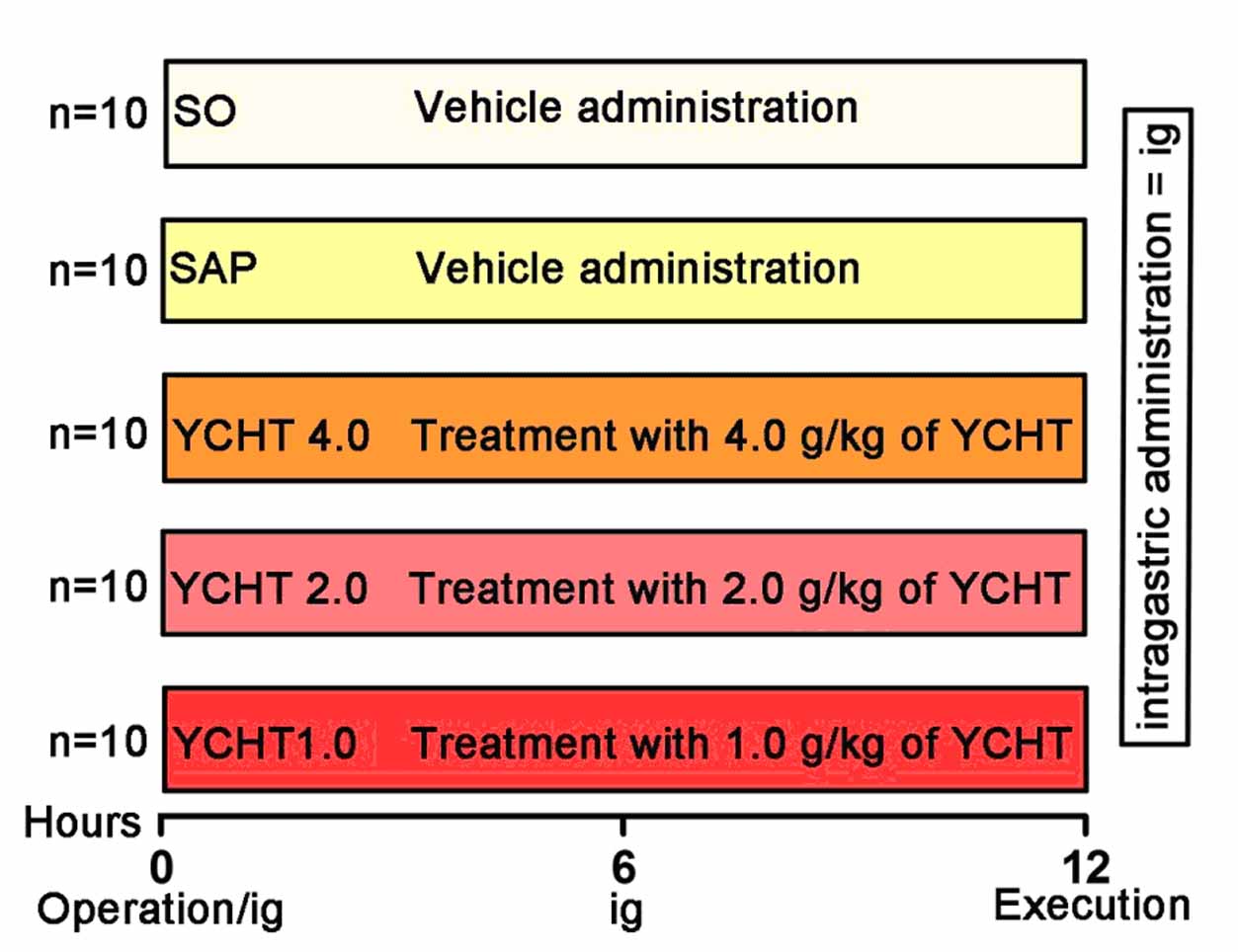

Supplement: FIGURE S2 — Experimental protocol of YCHT attenuates SAP. [file Image_2.JPEG]

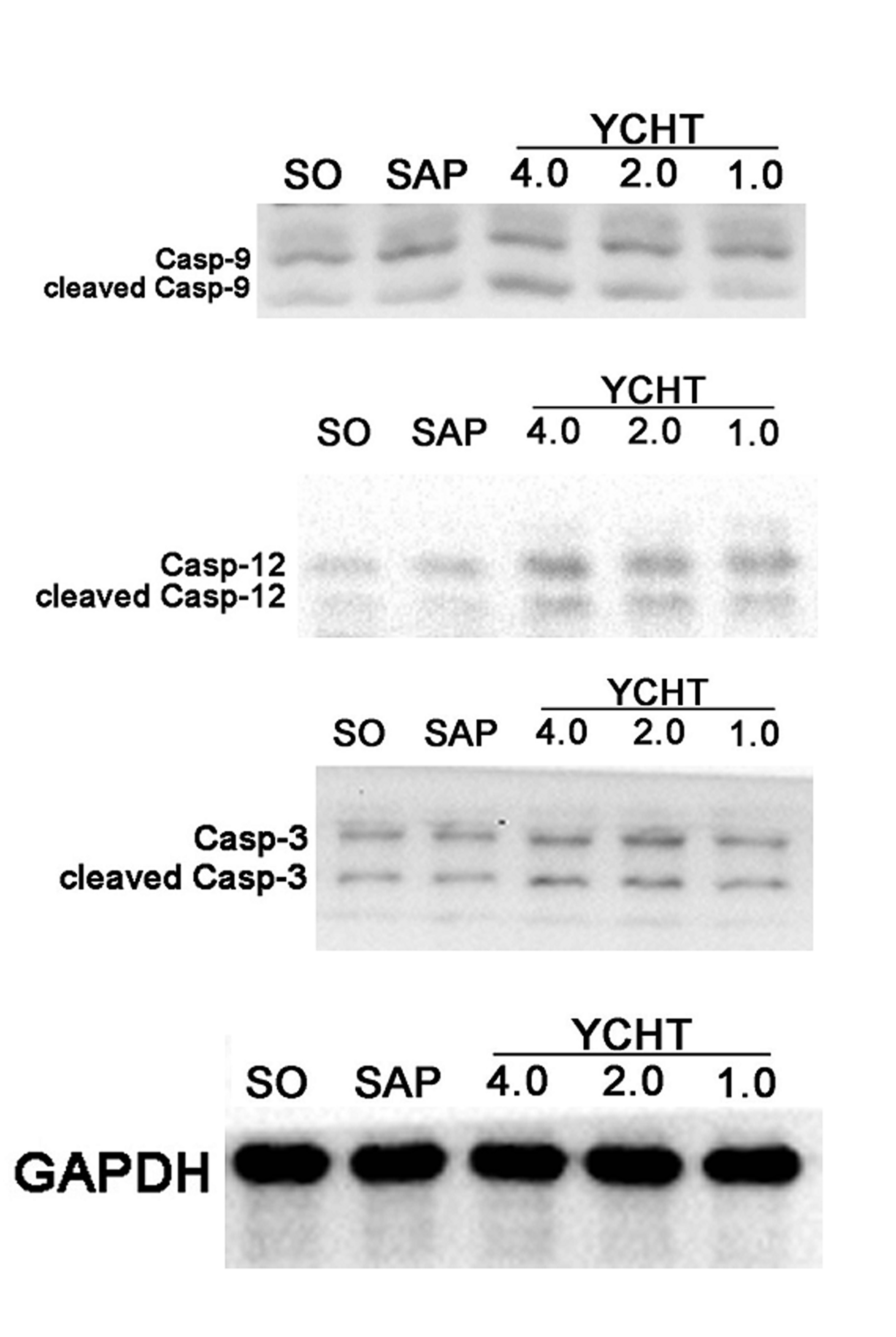

Supplement: FIGURE S3 — The original bands of western blot results in Figure 5. [file Image_3.JPEG]

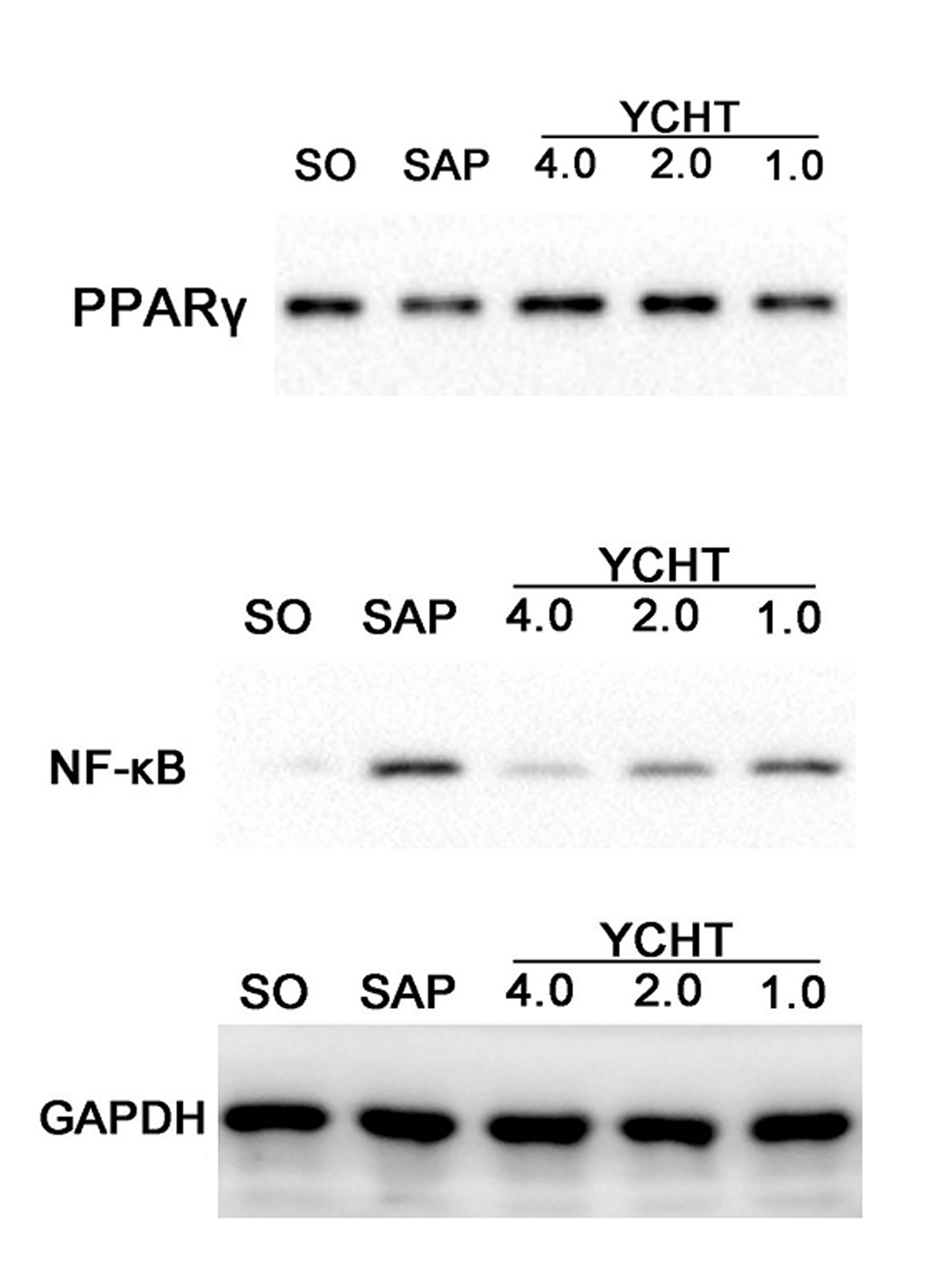

Supplement: FIGURE S4 — The original bands of western blot results in Figure 7. [file Image_4.JPEG]
